# Epidemiological analysis of the Kaohsiung city strategy for dengue fever quarantine and epidemic prevention

**DOI:** 10.1186/s12879-020-4942-y

**Published:** 2020-05-15

**Authors:** Chao-Ying Pan, Wei-Liang Liu, Matthew-P. Su, Te-Pin Chang, Hui-Pin Ho, Pei-Yun Shu, Joh-Jong Huang, Li-Jen Lin, Chun-Hong Chen

**Affiliations:** 1 Department of Health, Kaohsiung City Government, Kaohsiung City, Taiwan; 2 National Mosquito-Borne Diseases Control Research Center, National Health Research Institutes, Zhunan, Taiwan; 3 Department of Biological Science, Nagoya University, Nagoya, 464-8602 Japan; 4 Center for Diagnostics and Vaccine Development, Centers for Disease Control, Ministry of Health and Welfare, Taipei, Taiwan, Republic of China; 5 Bureau of Social Affairs, Tainan City Government, Tainan City, Taiwan; 6 National Institute of Infectious Diseases and Vaccinology, National Health Research Institutes, Zhunan, Taiwan

**Keywords:** Dengue virus, Imported dengue cases, Concealment, Indigenous dengue fever, Airport fever screening, Noncontact infrared thermometers

## Abstract

**Background:**

Dengue is endemic in over 100 countries and is an important public health problem worldwide. Dengue fever is not endemic in Taiwan; the importation of dengue viruses from neighboring countries via close commercial links and air travel is considered to be the cause of local outbreaks. Therefore, efforts toward disease control have focused on preventing the importation of dengue into Taiwan. In this study, we investigated the relationships between the numbers of imported and indigenous dengue cases to test the validity of this strategy.

**Methods:**

Data on cases of dengue fever that occurred between 2013 and 2018 were obtained from the surveillance systems of the Taiwan Center for Disease Control and Kaohsiung City Health Department. Standard epidemiological data, including the monthly numbers of indigenous and imported cases of dengue, were calculated. Potential associations between the numbers of indigenous and imported cases were investigated using correlation analyses.

**Results:**

We identified a possible relationship between the period of disease concealment and the number of imported dengue cases, which resulted in epidemics of indigenous dengue fever within local communities. Further analysis of confirmed cases during previous epidemics in Kaohsiung City found that the risk of indigenous dengue fever may be related to the likelihood that patients with imported dengue fever will stay within local communities.

**Conclusion:**

Given the correlations found between imported and indigenous cases of dengue fever, as well as the relationship between the disease concealment period and the risk of indigenous dengue fever, prevention of disease importation and efficient identification of dengue cases within high-risk communities remain the major priorities for disease control.

## Background

Dengue is the most prevalent emerging arthropod-borne viral infection (arbovirus) in humans worldwide and is highly endemic in many Western Pacific, Southeast Asian, and South American countries [1–3]. Transmitted via bites from infective female *Aedes* mosquitoes, dengue has rapidly spread in the past few decades, and this has been driven in part by climate change and increased rates of global trade and international travel [4–7]. Approximately 2.5 billion individuals are currently at risk of contracting dengue, and approximately 100 million clinically manifesting dengue infections are estimated to occur worldwide on an annual basis [8, 9]. Disease symptoms vary significantly and range from asymptomatic cases to classic dengue fever (DF), as well as more serious complications (e.g., dengue hemorrhagic fever or dengue shock syndrome) [10].

The *dengue virus* (DENV) itself is an enveloped virus with a single-stranded, positive-sense RNA genome, belonging to the genus *Flavivirus* of the family *Flaviviridae*. DENV contains 5′- and 3′-untranslated regions and a single open reading frame encoding a single polyprotein that can be cleaved into three structural proteins (capsid, premembrane/membrane, and envelope) and seven nonstructural proteins (NS1, NS2A, NS2B, NS3, NS4A, NS4B, and NS5) [11, 12]. There are four genetically and antigenically distinct DENV serotypes, recognized as DENV-1, − 2, − 3, and − 4 [13]. Since 2006, southern Taiwan has faced dengue outbreaks of varying magnitudes annually, with relatively large outbreaks occurring in 2014 and 2015; DENV-1 and -2, respectively, were identified as the major serotypes in these cases [14].

Transmission of dengue in Taiwan is driven by two species of *Aedes* mosquitoes: *Aedes albopictus* and *Aedes aegypti*. The former is found throughout Taiwan, while the latter is restricted to the south [15]. Indigenous dengue is not considered endemic in Taiwan; thus, importation of DENV (due to close commercial ties and human migration from neighboring Southeast Asian countries) is thought to be the underlying cause of DENV outbreaks. Preventing the importation of disease has therefore become the major objective of quarantine for dengue fever and the epidemic prevention network in Taiwan.

The emergence of Severe Acute Respiratory Syndrome in 2003 highlighted the role of international travel in the rapid spread of infectious diseases and prompted many countries to establish border control strategies and use noncontact infrared thermometers (NCITs) at international airports to reduce the risk of imported infections [16, 17]. Taiwan expanded this noninvasive diagnostic tool to screen for various infections, including dengue, at all major entry borders.

Such strategies to reduce the risk of disease importation are particularly relevant for Kaohsiung City, a major city in the south of Taiwan, as it was the main site of the 2014 and 2015 dengue outbreaks [18, 19]. Constant surveillance of dengue seroprevalence and the use of airport fever screening systems to test individuals suspected of having dengue fever have become central to the disease control program of the city.

However, the relationship between the numbers of indigenous and imported cases remains unclear [20]. This relationship is complicated in part by cases of asymptomatic disease and prolonged periods of disease latency, which can result in secondary/tertiary waves of infection many weeks after the first wave [3]. In theory, the latency period in mosquitoes is between 8 and 12 days, while the maximal latency in humans is between 3 and 8 days [10]. As such, the second wave of infections typically occurs 2–3 weeks later, and the third wave can occur 4–6 weeks after the initial bout of infections. Detailed case reports collected over prolonged periods are required to dissect the complex interactions between indigenous and imported cases of dengue fever.

In this study, we investigated the number of indigenous and imported cases of dengue fever in Kaohsiung City between 2013 and 2018, including the country of importation and status/occupation of the infected individual. We also mapped out the monthly number of cases confirmed by the Taiwan Center for Disease Control (CDC) surveillance system throughout this period. We subsequently tested for correlations between indigenous and imported cases, testing both the number of cases and the month in which they were reported. We found a significant correlation between the two types of infection after accounting for the disease latency period and delays in case reporting. Our results suggest that importation of the virus is indeed driving outbreaks of dengue fever in Taiwan, indicating that disease prevention strategies should continue to focus on human migration patterns and the efficient identification of dengue fever to reduce the incidence of disease.

## Methods

### Database and case definitions

We identified all cases of dengue fever reported between January 2013 and September 2018 in the public databases of the Taiwan CDC (http://www.cdc.gov.tw/english/index.aspx) and the Kaohsiung City Health Department. We used the definitions of dengue set by the Taiwan CDC [21]. As such, according to the Diagnostic Center of the Taiwan CDC, a confirmed case of dengue fever requires positive detection of RNA using real-time reverse transcriptase polymerase chain reaction and/or serological diagnosis through an IgM/IgG enzyme-linked immunosorbent assay [22]. A standard protocol was adopted by local physicians and epidemiologists to collect individual-level information on each dengue fever case, including the onset of dengue illness, age, gender, clinical manifestations, reporting hospital, diagnosis results and travel history. Dengue case collection and surveillance involved both passive (hospital-based reporting systems) and active (fever screening at airports, self-reporting, expanded screening for contacts of confirmed cases, patients with fever of unknown origin and school-based reporting) detection.

An imported case of dengue fever is defined as a confirmed case in a patient who had traveled to another country, in which dengue fever is endemic, within 2 weeks prior to the onset of illness. Indigenous cases are defined as cases reported in infected individuals without any travel history. The total number of imported cases of dengue included inbound passengers diagnosed as dengue-positive via airport screening methods and those diagnosed in clinics or hospitals following entry into the local community.

### Surveillance of dengue

The airport fever screening program was introduced in 2003 to detect travelers infected with Severe Acute Respiratory Syndrome, dengue, or other diseases. In this study, we analyzed data collected between January 2013 and September 2018 from the International Kaohsiung Airport. All airline passengers (outbound and inbound) were requested to have their body temperature measured using NCITs. Two thermal NCITs were set up at each entry gate, with each NCIT having an infrared-thermal camera. Travelers with a detected temperature > 37.5 °C were rechecked by quarantine officers with a symptoms survey and assessed using an ear thermometer. Travelers with a temperature > 38 °C, who had arrived from a dengue-affected area, were triaged for additional diagnostics. Specimens were obtained for testing with the Dengue NS1 Rapid Test (SD BIOLINE Inc., Korea; sensitivity and specificity estimated at 92 and 98%, respectively) at an airport inspection station. Duplicate specimens were sent to the central laboratory of the Taiwan CDC for simultaneous confirmation through molecular and/or serological diagnosis.

### Statistical analysis

All statistical analyses were conducted using the SPSS Statistics software, version 22 (IBM Corp., Armonk, NY, USA). Overall mean scores were calculated and compared using paired t-tests. Potential correlations between the numbers of indigenous and imported cases were analyzed using Pearson’s correlation coefficient test and two-way analysis of variance. A *p*-value < 0.05 was considered statistically significant.

## Results

### Statistical analysis of indigenous and imported cases of dengue fever in Kaohsiung City between 2013 and September 2018

#### Statistics for the total number of imported cases of dengue fever in Kaohsiung City

A total of 239 imported cases of dengue (32, 44, 61, 37, 34, and 31 cases in 2013, 2014, 2015, 2016, 2017, and 2018, respectively) were identified in Kaohsiung City during the examination period (Table 1), including 80 women (33.5%) and 159 men (66.5%). The majority of cases involved individuals arriving from Southeast Asian countries: 48 from the Philippines (20.0%), 47 from Indonesia (19.6%), 33 from Vietnam (13.8%), 30 from Malaysia (12.6%), 24 from Thailand (10.0%), and 13 from Singapore (5.4%). Additional cases were also imported from the Indian subcontinent (i.e., India, Bangladesh, Sri Lanka, and the Maldives) and the South Pacific region (Tuvalu). Supplementary Fig. 1 shows the top and bottom 5 countries from which dengue was imported, and the distance from these countries to Kaohsiung.

**Table 1:** Countries from which confirmed dengue cases were imported from between 2013 and September of 2018

Country	2013	2014	2015	2016	2017	2018	Total
Philippines	7	7	11	12	4	7	48
Indonesia	12	6	17	5	2	5	47
Vietnam	2	3	5	5	12	6	33
Malaysia	0	8	8	4	5	5	30
Thailand	4	4	6	4	3	3	24
Singapore	1	3	5	3	1	0	13
China	1	10	1	0	0	0	12
Cambodia	0	1	2	2	1	0	6
Maldives	0	0	2	1	0	3	6
Myanmar	1	0	1	1	2	0	5
Sri Lanka	3	0	0	0	1	1	5
Australia	0	0	2	0	0	0	2
India	0	0	0	0	1	1	2
Laos	1	0	0	0	0	0	1
Bangladesh	0	0	0	0	1	0	1
Marshall Islands	0	0	0	0	1	0	1
Tuvalu	0	1	0	0	0	0	1
Japan	0	1	0	0	0	0	1
South Africa	0	0	1	0	0	0	1
Total	32	44	61	37	34	31	239

**Fig. 1:**
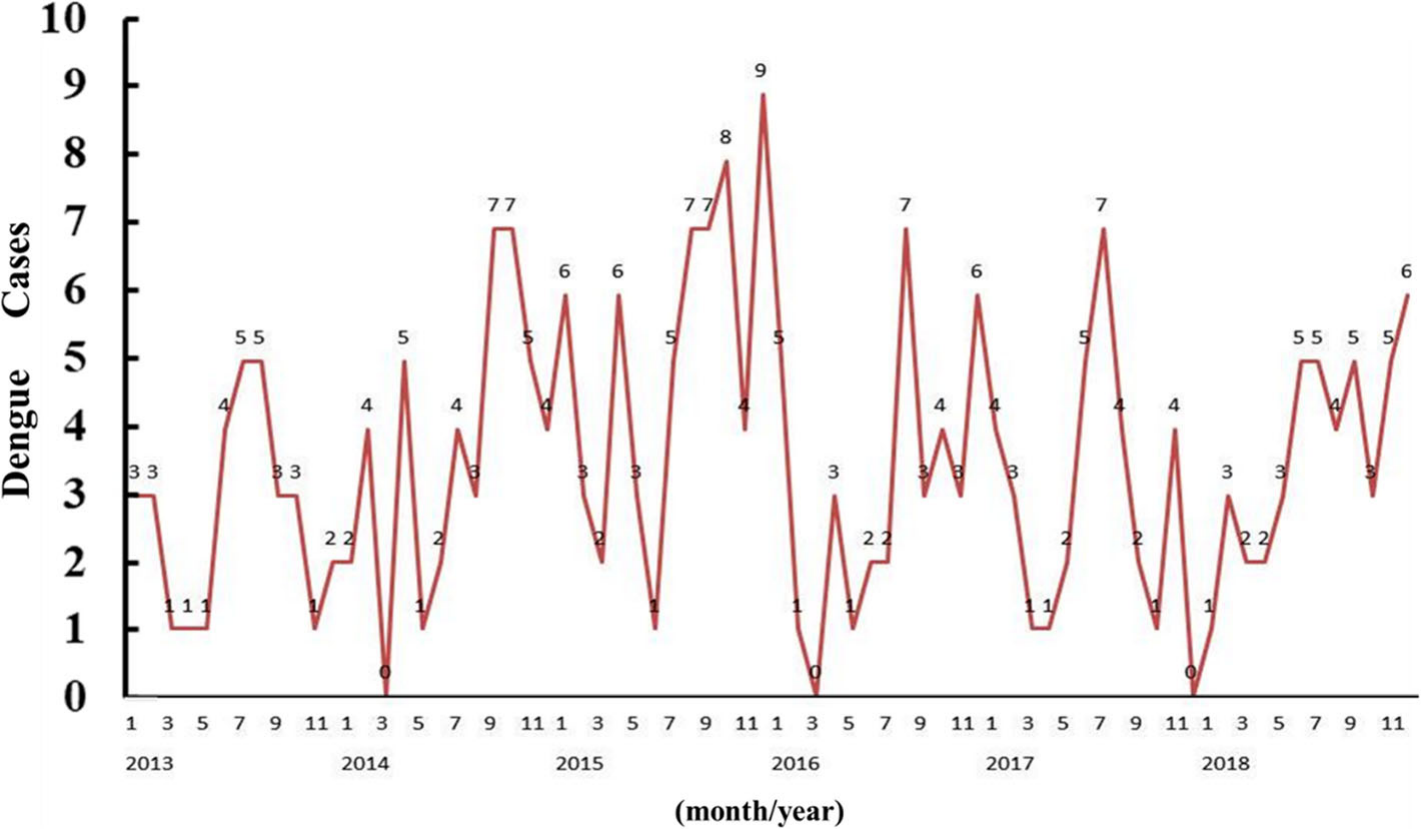
Fluctuations in the number of dengue cases from 2013 to September 2018. The cases were recorded by the Taiwan CDC surveillance system in Kaohsiung City from 2013 to September 2018. Monthly data are shown to reflect imported cases for all years

Further analyses of the 239 imported cases of dengue revealed that 142 of the infected individuals (59.4%) were Taiwanese nationals who had traveled abroad. However, foreign workers and tourists entering the country accounted for a further 15.5 and 8.8% of cases, respectively (Table 2). Analysis of patient occupation is presented in Table 3, with home management, business, and studying being the most commonly cited occupations (together accounting for 46.4% of cases). Figure 1 shows fluctuations in the monthly reporting rates of imported cases of dengue in Kaohsiung City (Fig. 1).

**Table 2:** Status of infected individuals suffering from imported dengue in Kaohsiung city from 2013 to September 2018

Status	2013	2014	2015	2016	2017	2018	Total
Travel abroad migration (business or study tour)	21	28	37	17	22	17	142
Foreign workers/labor	3	4	11	8	4	7	37
Foreign tourists	1	3	3	8	3	3	21
Returning overseas Chinese	1	6	2	2	0	0	11
Foreign student	0	3	5	0	1	0	9
Foreign crew/fishermen	1	0	2	1	3	1	8
New immigrants (including family)	5	0	1	1	1	3	11
Total	32	44	61	37	34	31	239

**Table 3:** Occupations of infected individuals suffering from imported dengue in Kaohsiung city from 2013 to September 2018

Occupation	2013	2014	2015	2016	2017	2018	Total
Home management	4	12	17	6	2	2	43
Business	5	10	4	6	5	5	35
Student	4	5	12	4	3	5	33
Manufacturing	0	3	9	2	6	6	26
Technical service industry	1	1	5	5	4	5	21
Healthcare industry	5	3	3	4	2	0	17
Teacher	5	3	0	1	0	1	10
Fisherman or crew	1	0	2	1	3	2	9
Construction industry	2	1	1	1	1	0	6
Finance and insurance	0	3	1	1	0	0	5
Transportation and communications	0	0	1	0	2	2	5
Religious/cultural service industry	1	0	1	0	2	0	4
Catering service industry	0	0	0	3	0	1	4
Tourism service industry	1	1	0	1	0	0	3
Human agency	0	0	0	2	0	1	3
Food processing industry	0	0	1	0	2	0	3
Civil service	0	0	3	0	0	0	3
Fishery/aquaculture	1	1	0	0	1	0	3
Wholesale and retail trade	1	0	0	0	1	1	3
Armed forces	1	1	0	0	0	0	2
Real estate and leasing industry	0	0	1	0	0	0	1
Total	32	44	61	37	34	31	239

#### Predominant serotypes of annual indigenous cases of dengue fever in Kaohsiung City

**Fig. 2:**
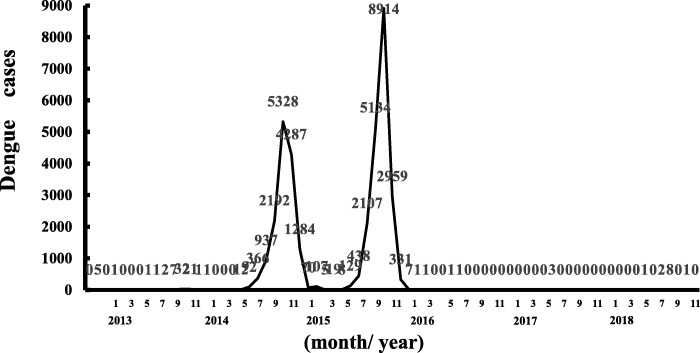
The number of indigenous cases of dengue in Kaohsiung City reported from 2013 to September 2018

Between 2013 and September 2018, there were 35,148 indigenous cases of dengue fever in Kaohsiung City, with 70, 14,999, 19,723, 342, 3, and 11 cases reported in 2013, 2014, 2015, 2016, 2017, and 2018 (up to the end of September), respectively. Primary cases of dengue fever were concentrated in the 24 consecutive months between May 2014 and April 2016 (Fig. 2). During this period, there were 35,062 indigenous cases of dengue fever in Kaohsiung City (99.8%). The major epidemic serotype of dengue fever in 2014 was serotype 1, while that from 2015 to April 2016 was serotype 2.

#### Concealment of cases for more than 3 days

Further analysis of the 239 confirmed imported cases revealed that 37 infected individuals did not stay within local communities after contracting dengue. This included 5/32 cases that occurred in 2013 (15.6%); 3/44 cases in 2014 (6.8%); 1/61 cases in 2015 (1.7%); 10/37 cases in 2016 (27.0%); 12/34 cases in 2017 (35.3%); and 6/31 cases in 2018 (19.4%) (Table 4).

**Table 4:** Numbers of confirmed cases of dengue that remained within local communities in Kaohsiung city from 2013 to September 2018

	2013	2014	2015	2016	2017	2018	Total
**Number of confirmed imported cases of dengue fever**	32	44	61	37	34	31	239
**Number of confirmed imported cases of dengue fever which did not staying in the community**	5	3	1	10	12	6	37
**Dengue fever imported cases into sthe community which were concealment for more than 3 days**	24	32	39	22	14	8	139

**Fig. 3:**
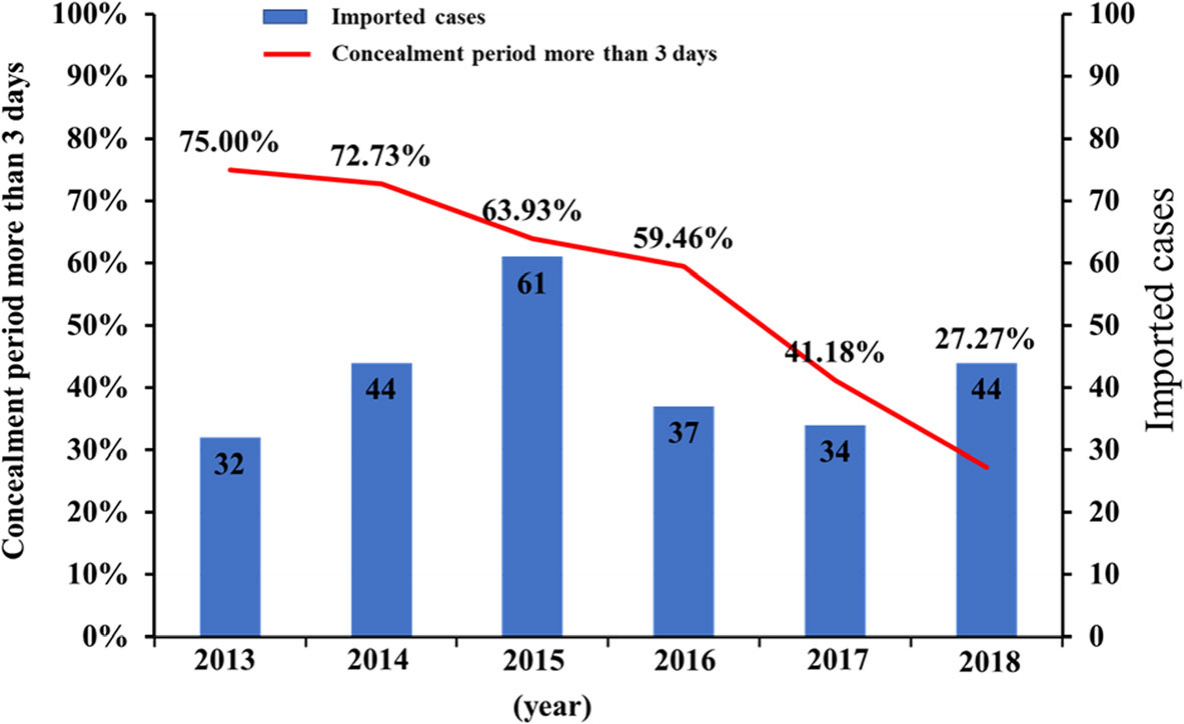
Analysis and correlations between the imported cases of dengue and concealment period. The number of imported cases of dengue and the percentage of cases with a concealment period > 3 days in Kaohsiung City reported from 2013 to September 2018

We also identified a significant number of cases whose concealment period was > 3 days (139/239, 58.16%). Of these, we found 24 such cases in 2013 (75.0% of cases that year), 32 in 2014 (72.7%), 39 in 2015 (63.9%), 22 in 2016 (59.5%), 14 in 2017 (41.2%), and 8 in 2018 (19.4%) (Fig. 3).

### Statistical analysis of the number of indigenous and imported cases of dengue fever in Kaohsiung City between 2013 and September 2018

The 35,148 confirmed cases of indigenous dengue fever reported in Kaohsiung City between 2013 and September of 2018, along with the 239 imported cases, were sorted according to the month of identification (*n* = 69). A significant positive correlation (paired sample correlation coefficient, r = 0.407, *p* = 0.001) was found between the numbers of imported and indigenous cases reported each month (Table 5).

**Table 5:** Statistical comparisons of indigenous and imported cases of dengue

**Paired sample statistics**								
		Average	N	Standard deviation	Mean value of standard error			
Group 1	Number of indigenous cases of dengue fever	509.39	69	1534.868	184.776			
	Number of import cases of dengue fever	3.42	69	2.11	0.254			
**Paired sample correlation**								
		N	correlation	signification				
Group 1	Number of indigenous cases of dengue fever	69	0.407	0.001				
**Verification of paired sample**								
**Paired difference number**								
	Average	Standard deviation	Standard error	Confidence interval of average 95%differene				
			Average	minimums	maximums	T	df	Significance (two tailed)
Number of indigenous cases of dengue fever	505.971	1534.009	184.673	137.462	874.48	2.74	68	0.008
Number of import cases of dengue fever								

However, actual clinical surveillance reports were delayed by approximately 1–2 months during local epidemics of dengue fever; furthermore, a concealment period ≥2 days was common. Therefore, instead of investigating correlations between reported cases in the same month, we tested for relationships between the number of imported cases in a single month and the number of indigenous cases reported 2 months later, also accounting for differences in the concealment period. When accounting for concealment periods > 2 days, we found significant positive correlations between the number of imported cases and the number of indigenous cases reported 2 months later (Pearson’s correlation coefficient, r = 0.459, *p* = 0.0001), > 3 days (r = 0.394, *p* = 0.001), and > 4 days (r = 0.387, *p* = 0.001) (Table 6).

**Table 6:** Correlations between the numbers of indigenous and imported cases of dengue

**Correlation**					
		Number of indigenous cases of dengue fever	Number of imported cases of dengue fever with a concealment period of longer than 3 days	Number of imported cases of dengue fever with a concealment period of longer than 2 days	Number of imported cases of dengue fever with a concealment period of longer than 4 days
Number of indigenous cases of dengue fever	Pearson correlation	1	Significance (two tailed)	0.459**	0.387**
	Significance (two tailed)		0.001	0.0001	0.001

**Correlation is significant on the 0.01 layer (two-tailed)

## Discussion

Dengue fever spreads via “virus-mosquito-human” transmission. Current preventive strategies focus on the mosquito itself, with a particular focus on environmental rectification and maintenance (e.g., targeting sites of mosquito breeding) [23, 24]. The large-scale outbreaks of dengue fever that occurred in Kaohsiung City between 2014 and 2015, which were likely the result of imported cases of dengue, showed the limitations of this vector-focused approach.

The spread and control of infectious diseases are key aspects in promptly preventing the occurrence of disease. Kaohsiung City experienced a severe epidemic outbreak of dengue fever from 2014 to 2015, in part due to a lack of real-time reporting of cases, which allowed the virus to spread in the community. Following this, the Kaohsiung City Government refocused its disease prevention efforts on border quarantine to prevent importation of disease, which should consequently reduce the number of indigenous cases resulting from secondary infections. The Kaohsiung City Government devised the following action plans to improve the anti-epidemic capacity of Kaohsiung City to prevent dengue fever. These efforts included the establishment of a quarantine referral station, a quarantine period for foreign workers and fishermen, return of new residents and foreign students to their home country for health care measures, and increased incentives for the local or returning citizens related to the prevention of epidemics.

During the 2014 and 2015 outbreaks, there were 14,999 and 19,723 cases of indigenous dengue fever, respectively. Case reports from early 2016 showed signs of a continuation of the 2015 epidemic, with 340 indigenous cases reported before April. However, there were only two cases of native dengue fever throughout the remainder of 2016. In 2017 and 2018, Kaohsiung City achieved the two lowest numbers of indigenous cases on record (3 and 11 cases, respectively) [19]. This significant reduction in the number of dengue cases was in part due to a complete reorganization of the dengue quarantine and epidemic preventive network in Kaohsiung in April 2016, though other factors (such as the emergence of short-lived cross-protection between heterologous Dengue serotypes following disease outbreaks) may have also contributed to the extent of this reduction.

A new project focusing on imported cases was initiated in July 2016 to cover both border quarantine and epidemic prevention work. In October 2016, the only “Quarantine referral station” in Taiwan was established at the Kaohsiung International Airport to identify infected travelers. Thus, the airport and seaport of Kaohsiung City constitute the front line of the border quarantine network in South Taiwan for the prevention of dengue fever epidemics.

Kaohsiung City possesses large native populations of *Aedes aegypti* and *Aedes albopictus*, resulting in a high risk of secondary dengue infection after importation of an initial case from abroad. Although the ongoing collection and testing of thousands of human and mosquito specimens suggest that the DENV has not yet localized in Kaohsiung, it is vital that control programs targeting mosquitoes continue to reduce the likelihood of future localization and minimize the risk of secondary outbreaks following the initial wave of infections [25]. Efforts for the control of dengue, whether focused on vector control or prevention of disease importation, are costly both in terms of resources and workload. Constant innovation is required to maintain the low number of dengue cases reported in Kaohsiung City, while reducing unnecessary expenditure. This includes improved, efficient targeting of high-risk individuals to reduce the likelihood of transmission throughout local communities. For example, foreign workers and fishermen from countries in which dengue is endemic could be screened for dengue (NS1) within the first 3 days of entering Taiwan [26]. Those identified as being dengue-positive would then receive hospital inpatient care. This approach will assist in isolating the DENV carrier from the vector mosquitoes in the community, effectively blocking DENV from spreading into the community from foreign sources. Furthermore, the use of mathematical modelling, both to predict patterns of future disease outbreaks and to test the origin of disease cases, has becoming increasingly widespread in recent years [27, 28]. The wealth of data available as a result of the dengue outbreaks in Kaohsiung could be utilized to develop such models and thus assist in disease control efforts.

Based on the above findings, the lowest record of local confirmed cases of dengue fever in Kaohsiung City in the past few years was the result of its unique geographical location used to strengthen the efficiency of quarantine outside the battle zone, optimize medical network resources against dengue, construct specific quarantine and epidemic prevention networks, and effectively shorten the incubation period of cases. In other words, the dengue protection network has successfully utilized the natural advantages of Taiwan for disease control to adopt an effective quarantine strategy. In spite of this, the threat posed by dengue (as well as other communicable diseases) is likely to increase in line with increases in international exchange and trade. Local networks must be able to constantly innovate and adopt an international focus to effectively combat dengue and prevent future outbreaks of dengue in Taiwan.

## Conclusions

Though there may be several factors influencing disease transmission (including vector migration), dengue fever outbreaks in Kaohsiung are the likely the result of imported cases of the disease, in which dengue virus is transported to Kaohsiung via infected individuals and then spread within the community. Therefore, it is vital that strategies, including modern quarantine methodologies and high-efficiency health care, are utilized to reduce the likelihood of dengue fever becoming endemic in Kaohsiung City.

## Supplementary information

**Additional file 1 Supplementary Fig. 1**. Map showing the distance between the top/ bottom 5 countries from which Dengue was imported and Kaohsiung city. The analysis chart was created after analyzing all collected imported dengue fever data from 2013 to 2018 and referring to the dengue map constructed by the Taiwan CDC (https://cdcdengue.azurewebsites.net/Imported.aspx). The world map is publicly available via OpenStreetMap (https://www.openstreetmap.org/).

## Data Availability

Information on all dengue cases identified from 2013 to 2018 is publicly available via the Taiwan Centers for Disease Control (http://www.cdc.gov.tw/english/index.aspx) and Taiwan Government Open Data (http://data.gov.tw/en) websites. Annotated datasets used and/or analyzed during this study are also available from the corresponding author on request.

## Abbreviations

CDC: Centers for Disease Control
DENV: Dengue virus
DF: Dengue fever
DHF: Dengue hemorrhagic fever
DSS: Dengue shock syndrome
NCIT: Noncontact infrared thermometer
SARS: Severe Acute Respiratory Syndrome
