# Supplementary material for: Epidemiological analysis of the Kaohsiung city strategy for dengue fever quarantine and epidemic prevention
Source: BMC Infect Dis. 2020 May 15;20:347. doi: 10.1186/s12879-020-4942-y (PMC7226716; doi:10.1186/s12879-020-4942-y)
Supplement: Supplementary file 1 — Additional file 1 Supplementary Fig. 1. Map showing the distance between the top/ bottom 5 countries from which Dengue was imported and Kaohsiung city. The analysis chart was created after analyzing all collected imported dengue fever data from 2013 to 2018 and referring to the dengue map constructed by the Taiwan CDC (https://cdcdengue.azurewebsites.net/Imported.aspx). The world map is publicly available via OpenStreetMap (https://www.openstreetmap.org/). [file 12879_2020_4942_MOESM1_ESM.docx]

**Supplementary Figure 1.**


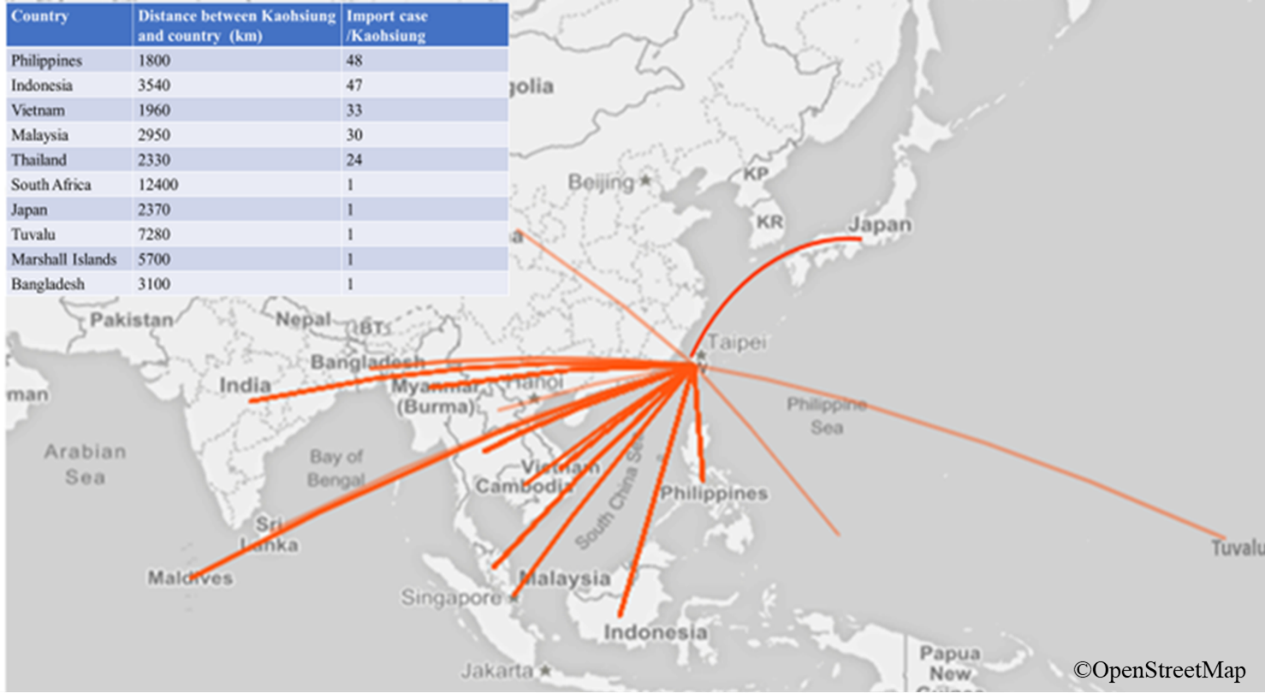


**Supplementary Figure 1. Map showing the distance between the top/ bottom 5 countries from which Dengue was imported and Kaohsiung city.** The analysis chart was created after analyzing all collected imported dengue fever data from 2013 to 2018 and referring to the dengue map constructed by the Taiwan CDC (<https://cdcdengue.azurewebsites.net/Imported.aspx>). The world map is publicly available via OpenStreetMap (<https://www.openstreetmap.org/>).
